# Testing the top-down feedback in the central visual field using the reversed depth illusion

**DOI:** 10.1016/j.isci.2025.112223

**Published:** 2025-03-15

**Authors:** Li Zhaoping

**Affiliations:** 1 University of Tübingen, Max Planck Institute for Biological Cybernetics, Tübingen, Germany

**Keywords:** Neuroscience, Sensory neuroscience, Cognitive neuroscience

## Abstract

In a new framework to understand vision, an information bottleneck impoverishes visual input information downstream of the primary visual cortex along the visual pathway; to aid ongoing visual recognition given the bottleneck, feedback from downstream to upstream visual stages queries for additional information. According to the central-peripheral dichotomy theory, this feedback is primarily directed to the central, rather than the peripheral, visual field. Counterintuitively, this theory predicts illusions visible only in the peripheral visual field, which lacks the feedback query to veto the illusions arising from misleading and impoverished feedforward signals. A paradigmatic example is the predicted and confirmed reversed depth illusion in random-dot stereograms. This theory further predicts that disrupting the feedback renders this illusion visible in the central visual field. We test and confirm this prediction using visual backward masking to disrupt the feedback. This feedback privilege for the central visual field underpins visual understanding through analysis-by-synthesis.

## Highlights

• An information bottleneck in vision makes visual perception vulnerable to illusions
• Feedback in visual pathway queries for more information, vetoing the illusions
• The peripheral visual field lacks this feedback, sees the reversed depth illusion
• Backward masking disrupts the feedback, making the illusion visible in the central visual field

## Graphical abstract

**Figure:**
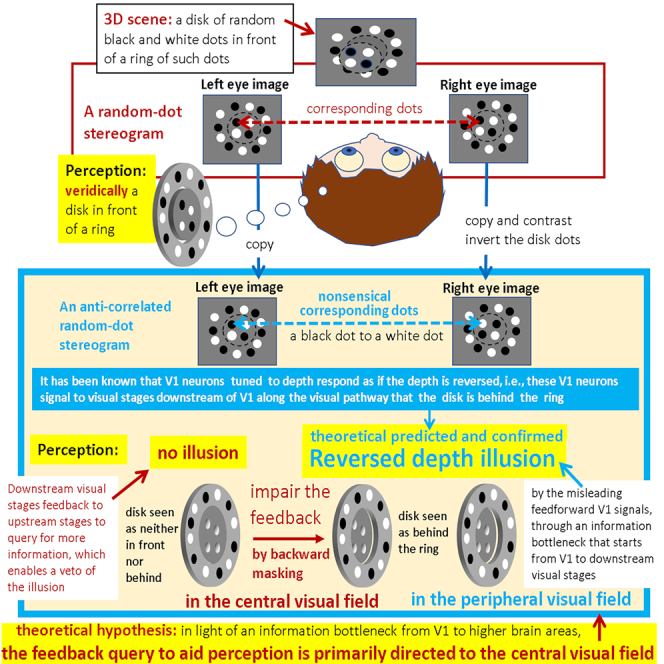

## Introduction

Perception is often viewed as a hypothesis about the sensory world, and perceptual inference as the process of hypothesis testing. Accordingly, sensory information is fed forward along the sensory (e.g., visual) pathway, suggesting initial hypotheses about the sensory scene; downstream stages along the pathway test these hypotheses to reach the final perceptual outcome. Many researchers have proposed that hypothesis testing is partly carried out by top-down feedback along the sensory pathway.^1^^,^^2^^,^^3^^,^^4^^,^^5^^,^^6^^,^^7^^,^^8^^,^^9^ The central-peripheral dichotomy (CPD) theory uniquely hypothesizes that this feedback for object recognition is stronger in the central than in the peripheral visual field.^10^ This paper presents a test of this theory by its falsifiable predictions. We use the terms “central vision” and “peripheral vision” to refer to vision in the central and the peripheral visual fields, respectively.

**Figure 1:**
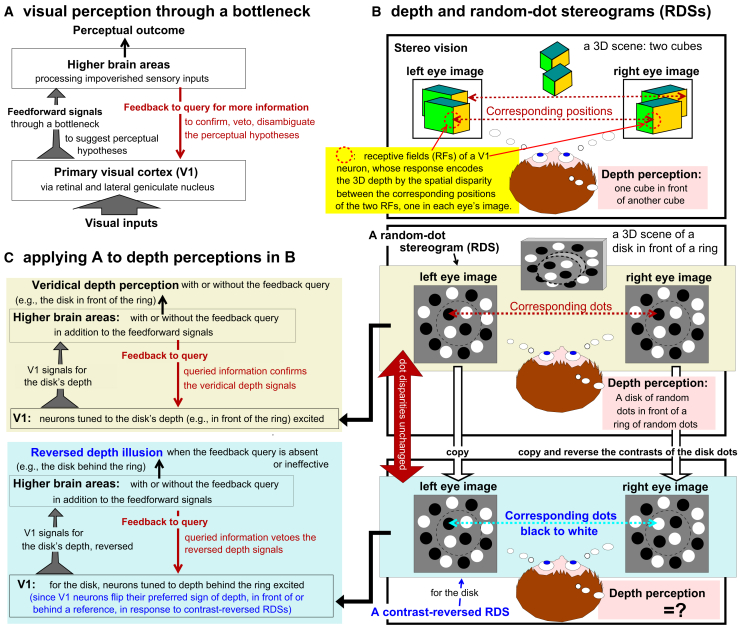
Visual perception through an information bottleneck and its application to depth perception in random-dot stereograms (RDSs) (A) A bottleneck starts from V1’s output to downstream stages along the visual pathway. The impoverished feedforward information suggests initial perceptual hypotheses. Feedback from downstream to upstream stages queries for additional information to confirm, veto, or disambiguate between the initial hypotheses to reach the perceptual outcome. (B) Stereo vision and RDSs. The depths of 3D locations are encoded by the responses of V1 neurons whose receptive fields (RFs) cover corresponding monocular locations of 3D object features. V1 neurons are activated by 3D features at their preferred depths, near or far (relative to fixation). A normal (contrast-matched) RDS depicts 3D surfaces, e.g., a disk in front of a ring, covered by random black and white dots. Each monocular image has no depth cues; the dashed curves are for illustration only and are not shown to viewers. For (e.g.) the disk, a contrast-reversed RDS is made by flipping the contrast polarity of every dot for this disk in just one (e.g., right) eye, so that a black dot in one eye corresponds to a white dot in the other eye. (C) V1 neurons are known to flip the sign (near or far) of their preferred depths in response to contrast-reversed RDSs. When only these V1 depth responses are fed forward, a contrast-matched RDS evokes a veridical depth percept, whereas a contrast-reversed RDS evokes a reversed depth illusion. A feedback query that solicits additional information from V1 can veto the illusion.

**Figure 2:**
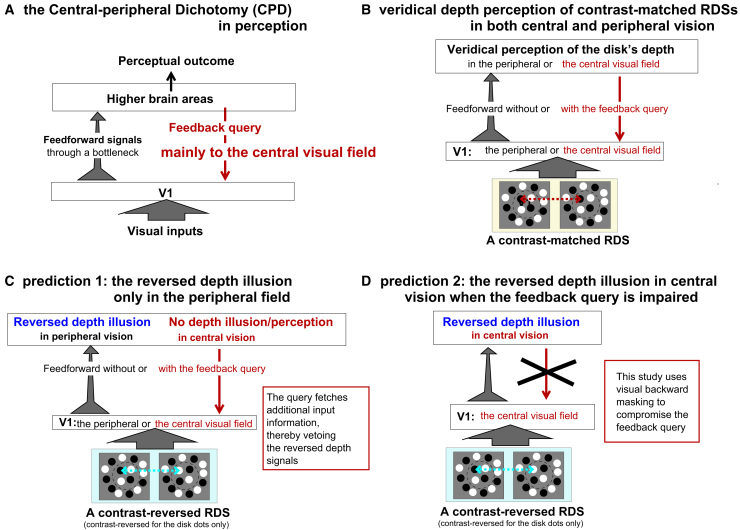
The CPD theory and its predictions for depth perception in the random-dot stereograms (RDSs) (A) The CPD theory hypothesizes that central, but not peripheral, vision is mainly for seeing (recognition) from the information that passes through the bottleneck. Thus, the top-down feedback that aids seeing by querying for additional information is mainly directed to the central visual field. (B) Applying the theory to depth perception of contrast-matched RDSs. With veridical depth signals from V1 responses to the RDS, both central and peripheral vision perceive veridical depth, with or without the feedback query. (C) As B, but for contrast-reversed RDSs. With the reversed depth signals from V1 responses, peripheral but not central vision sees the reversed depth. The additional information obtained by the feedback query in central vision vetoes this reversed depth illusion. (D) A prediction: reversed depth illusion in central vision when the feedback query is impaired.

The CPD theory is motivated by the information (or attentional) bottleneck that makes us blind to more than 99% of visual input information.^11^^,^^12^^,^^13^^,^^14^ It is also motivated by an early-selection idea that the bottleneck starts early at the output of the primary visual cortex (V1), so that visual inference downstream of V1 must proceed in light of this bottleneck.^10^ This early-selection idea is in turn motivated by the growing evidence for the V1 saliency hypothesis (V1SH), which posits that V1 creates a saliency map to guide selection by guiding gaze exogenously.^13^^,^^15^^,^^16^ Impoverished information through the bottleneck often makes recognition difficult. The CPD theory proposes that downstream stages use feedback to query for additional information from upstream stages such as V1 to aid ongoing recognition^10^^,^^17^ (Figure 1A), and that, to save brain resources, this feedback query is directed primarily to the central visual field (Figure 2A).

Unusual for theories of vision, the CPD theory has predicted non-trivial visual illusions that have been experimentally confirmed subsequently.^18^^,^^19^ Furthermore, these illusions are (as predicted) visible in the peripheral visual field only. This is because they arise from a lack of the feedback query when the feedforward visual input, impoverished by the bottleneck, is misleading. This paper reports the test of a natural further prediction: one such illusion, the reversed depth illusion, becomes visible in the central visual field when the feedback query is compromised. We begin by explaining the basis of this prediction.

A diagnostic information lost beyond V1 is about the eye of origin of visual input, since V1 is the only cortical area with a substantial number of monocular neurons^13^^,^^20^ to signal whether the left or right eye receives a monocular input. Humans are blind to eye-of-origin,^21^ even though depth perception by stereo vision requires this information at its initial processing stage. The information bottleneck starting from (though not necessarily ending at) V1 often makes perceptual outcomes ambiguous or even illusory. For example, too brief a glimpse makes it difficult to distinguish a red apple from a red rose. Our brain can use an internal model of the visual world to synthesize the detailed, would-be, sensory signals for each hypothesis or guess (e.g., a red apple) about the scene. By the proposed feedback query, the would-be and the actual neural responses at upstream visual stages such as V1 to the hypothesized scene (e.g., the apple) can be compared. A good or poor match between the would-be and actual upstream neural responses increases or decreases the likelihood of the hypothesis to become the final perceptual outcome.^10^ This process can veto an incorrect initial hypothesis to prevent an illusion when a poor match leads to a large decrease of the likelihood. By querying only the most useful information to resolve the perceptual ambiguity, the amount of information queried can be accommodated by the tight bottleneck. For example, to disambiguate between an apple and a rose, querying about object shape rather than color is likely more useful. However, the query requires a longer viewing duration.

We apply this framework to stereo depth perception (Figure 1B). Many V1 neurons are binocular with two receptive fields (RFs), one in each monocular image, tuned to similar image features.^13^ The preferred depth (of features in 3-dimensional (3D) space) of a neuron is determined by the preferred spatial disparity between the (matched sub-fields of the) two monocular RFs. Near or far objects activate neurons tuned to near or far depths (Figure 1B, top). A random-dot stereogram (RDS) can depict 3D surfaces covered by black and white dots randomly distributed on the surfaces. The disparity between the two dots in each pair of binocularly corresponding dots gives a depth cue for the underlying surface. Figure 1B (middle) schematizes an RDS for a disk in front of a ring using identically-sized dots to eliminate perspective cues. The dots for the disk or ring activate V1 neurons tuned to a nearer or farther depth. These V1 responses suggest to downstream stages a perceptual hypothesis for a disk in front of the ring. If the downstream stages feedback to query for additional information, the additional information should confirm this hypothesis. Hence, one perceives veridically a disk in front of the ring, with or without the query (Figure 1C, upper), in central or peripheral vision (Figure 2B).

If the contrast-polarities of the dots are flipped in one monocular image (without changing the dot positions) for one depth surface, e.g., the disk, a contrast-reversed, or anti-correlated, RDS is made (Figure 1B, bottom). For this disk, a black dot in one eye corresponds nonsensically to a white dot in the other eye. However, for reasons we explain later, V1 neurons preferring the disparity opposite to that of the contrast-reversed dots are excited, such that, e.g., a near disk now excites neurons tuned to far depth.^22^ These V1 responses report to downstream areas that the disk is behind the ring, potentially evoking a reversed depth illusion. However, if the feedback queries for additional information from V1, such as the responses from V1’s monocular neurons or other neurons that signal the contrast reversal of the corresponding dots, the illusion can be vetoed. Thus, the CPD theory predicts that this illusion should be visible in the peripheral visual field, which lacks the feedback query, but not in the central visual field, which has the feedback query^10^ (Figures 1C and 2C). This prediction is surprising since typically peripheral vision sees less and human observers have been reported as unable to see the reversed depth.^2^^,^^23^^,^^24^^,^^25^ Nevertheless, this prediction is confirmed,^18^ without contradicting the previous reports which examined only central vision.

A further prediction naturally follows: the reversed depth illusion becomes visible in central vision if the feedback query is impaired (Figure 2D). Falsifying this prediction would undermine the CPD rational behind this illusion in peripheral vision. This study tests and confirms this prediction. The feedback is compromised by backward masking, a technique in which a briefly presented visual image is replaced by another image, the mask, removing the relevant V1 responses from the query about the original image. Typically, when there is an ambiguity between alternative perceptual hypotheses, backward masking makes discrimination more difficult, because the feedback query (for additional information) to disambiguate is disrupted.^26^^,^^27^ When the dominant perceptual hypothesis—the reversed depth—arises from the misleading feedforward signals from the depth-tuned V1 neurons through the bottleneck, blocking the feedback query disables the veto, thereby making the illusion visible.

In the next section, we show by a main experiment that backward masking is achieved by dynamic RDSs made of successive RDS frames, Δt=10 ms per frame, so that each frame is masked by the next one. Across the frames, the disparity of the disk dots (with the ring dots always at zero disparity), the contrast-matching rule for the disk dots (contrast-matched or contrast-reversed), and the statistical properties of the disk and ring (e.g., sizes and positions) remain constant. Only the exact positions and colors (black and white) of the randomly generated dots on the disk and the ring vary between the frames. Indeed, the reversed depth illusion becomes visible in central vision by this backward masking. Then, we show in a secondary experiment that making Δt larger to, e.g., Δt=100 ms, would make the backward masking ineffective, and that a small Δt=10 ms could even enhance the visibility of the reversed depth illusion when the RDS is viewed at a more peripheral location with the center of the disk at an eccentricity of $10.1^{\circ }$, even though this illusion is already visible at this peripheral location in static RDSs (implying that, as will be discussed later, the feedback query, although much weaker such that it could not effectively veto the illusion in static RDSs, is not completely absent so that it could be further reduced by backward masking).

The main difference between this study and those in our previous study^18^ includes the following. First, the studies had different aims: the previous study tested the prediction that the reversed depth illusion exists only in peripheral vision, whereas the current study tests the prediction that disabling the feedback query allows the illusion to appear in central vision. Second, the current study includes RDS conditions with very small Δt values to evoke effective backward masking that impairs the feedback query, whereas the previous study used a Δt=100 ms, which was too large to compromise the feedback query. Third, the current study, by a secondary experiment, included stimulus conditions with different Δt=10,20,40,50,100 ms to explore the time δt in the feedback query process, whereas the previous study had only Δt=100 ms because it did not aim to explore δt. Fourth, only the previous study included the half-matched RDS conditions, in which half of the disk’s dots were binocularly contrasted-matched and the other half were contrast-reversed, as these conditions are less relevant to the current study. In addition, our current study is motivated by findings from a second previous study,^28^ whose aim was to probe the dynamics of the feedforward and the feedback processes for visual inference in central vision, rather than testing the CPD predictions. Compared to this second previous study,^28^ the current study differs not only in its goal, but also in (1) its focus on the reversed depth illusion and (2) its additions of Δt values (10, 40, and 50 ms) and the peripheral viewing conditions.

When a contrast-reversed stereogram contains only a few objects,^2^^,^^25^ human vision sees veridical depth. When contrast-reversed RDSs have sufficiently dense dots, many have reported that humans fail to see veridical depth or reversed depth^23^^,^^24^^,^^29^^,^^30^ (albeit examined only in central vision). This failure to see the reversed depth signals from V1^22^ was previously explained by the notion that V1 activities are not directly linked with perception.^31^ In retrospect, this failure was due to the use of static RDSs or dynamic RDSs with too large a Δt>50 ms.^24^^,^^29^ One study^32^ observed an effect of Δt when using four Δt values between 24 ms and 188 ms, prompting ideas about transient versus sustained processing channels for depth perception. However, the effect was weaker than it would be otherwise, and the data were hard to interpret, because the left and right eye inputs were asynchronous by about 12 ms by the use of stereo shutter glasses. Another study^30^ similarly used dichoptically asynchronous inputs, finding no reversed depth illusion in dynamic RDSs. In the CPD perspective, since peripheral vision sees the reversed depth even in static RDSs, it is a lack or a dysfunction of the feedback query, rather than a short Δt, that is essential for seeing the illusion.

## Results

**Figure 3:**
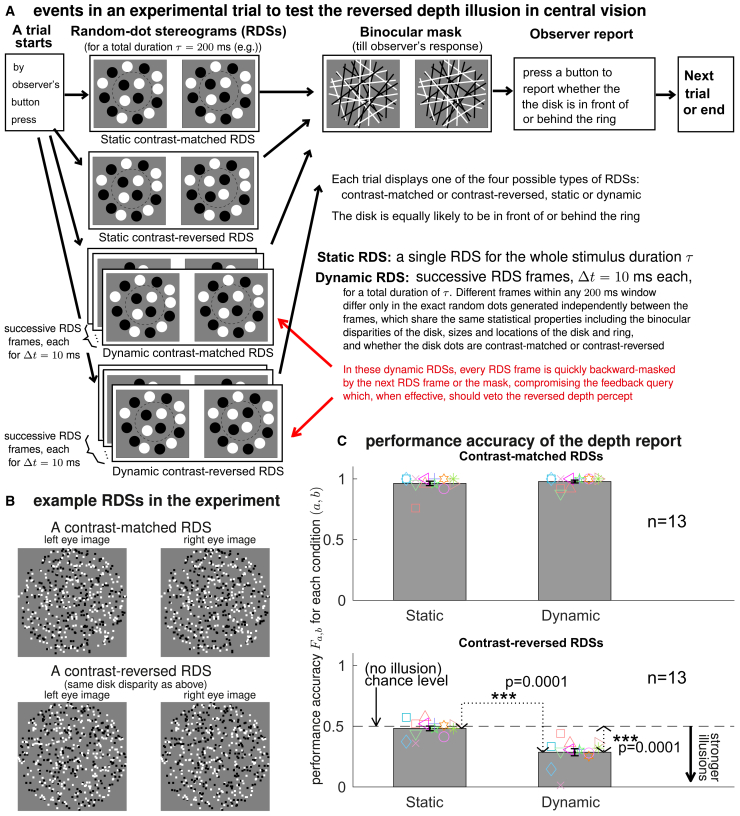
The main experiment: the reversed depth illusion appears in the central visual field when backward masking compromises the feedback query (A) Experimental design. Observers gave forced-choice reports on whether the central disk appeared in front of, or behind, the surrounding ring. The four stimulus conditions, static or dynamic, contrast-matched or contrast-reversed, were randomly interleaved. (B) Example contrast-matched and contrast-reversed RDSs, each has a disk with a positive disparity. (C) Task performance accuracy $F_{a,b}$ in each stimulus condition (a,b), defined as the ratio between the number of the condition (a,b) trials in which the depth report agreed with the disk’s disparity and the number of all the condition (a,b) trials, averaged across n=13 observers (one non-naive). For contrast-reversed RDSs, seeing the reversed depth illusion results in an accuracy smaller than the chance level of 0.5. The bars mark the averages across observers, with their standard errors marked by the error bars. Each observer’s accuracies are marked by a unique, colored, symbol across the four conditions (symbols across observers are horizontally dispersed somewhat in each condition to reduce clutter). An “∗∗∗” and the accompanying p value (p≤0.001, permutation test) are the result of a statistical, permutation, test for equivalence between the two average accuracies, or between an average accuracy and the chance level, connected by dotted black lines with arrowheads.

Two visual psychophysics experiments were carried out, in which observers gave forced-choice reports on whether the disk was perceived as in front of, or behind, the surrounding ring in the RDS. In the main experiment, observers viewed the RDS in the central visual field, and the RDS was dynamic or static, contrast-matched or contrast-reversed, as schematized in Figure 3. When the RDS was static, a single RDS frame was shown for a duration of τ, typically τ=200 ms. When the RDS was dynamic, successive RDS frames were shown, Δt=10 ms per frame, for a total duration of τ, and the random dots in different RDS frames within any 200 ms were independently generated given the disk and the ring. This experiment confirmed the prediction that, when the contrast-reversed RDSs were viewed in the central visual field, observers were only able to see the reversed depth illusion in dynamic RDSs. The secondary experiment extended the main experiment by adding dynamic RDS trials with Δt=20 ms, 40 ms, 50 ms, and 100 ms and adding trials in which the RDSs were shown at a more peripheral visual location. This experiment confirmed the previous findings (in a previous study^18^ and the main experiment) that the reversed depth is only visible at a more peripheral visual location when the RDS is static or when each RDS frame in a dynamic RDS was shown for a sufficiently long duration Δt. Additionally, the secondary experiment showed that the reversed depth illusion is more visible when Δt is smaller. Furthermore, it confirmed the prediction that, when Δt is sufficiently small, central and peripheral vision are statistically equivalent in seeing the illusion.

In both experiments, the total RDS viewing duration in a trial was τ=200 ms or a multiple of 200 ms, after which a binocular mask containing random black and white lines was shown (see Figure 3A). When τ>200 ms, the RDS stimuli in the first 200 ms were replayed in each subsequent 200 ms interval to allow sufficient viewing time for the RDS stimuli so that observers can do the task sufficiently well in contrast-matched trials. Note that making τ>200 keeps Δt unchanged in dynamic RDSs and keeps a static RDS static.

In addition to the viewing eccentricity of the RDS, a stimulus condition is specified by (a,b), with a=match or reversed and b=static or dynamic (or specified by the Δt value). The performance accuracy of an observer in condition (a,b) trials is defined by the fraction $F_{a,b}\equiv n/n_{total}$, in which $n_{total}$ is the number of the condition (a,b) trials performed by this observer, and n is the number of these trials in which the report on the disk’s depth relative to the ring agreed with the binocular disparities of the disk dots (relative to the ring’s dots). In analyzing the accuracy data, we used two-factor repeated (matched-sample) ANOVA (for the secondary experiment) and permutation tests, which are described in method details of the STAR Methods section.

### Unmasking the reversed depth illusion by backward masking in central vision

In each trial of the main experiment, observers viewed a static or dynamic RDS and gave a forced-choice report (by pressing a button) on whether the disk was in front of, or behind, the ring. The monocular images intended for the left and right eyes were displayed in the left and right halves of the screen of a Mitsubishi 21-inch cathode-ray tube (CRT). A stereoscope used four mirrors to show these images to the left and right eyes. The arrangement of the CRT and the stereoscope (purchased from Cambridge research systems) was identical to that in these previous studies.^17^^,^^18^^,^^28^^,^^33^ Each monocular image had a constant gray background. A black binocular rectangular frame enclosed the ring and disk regions. This frame was constantly presented throughout an experimental session, serving to anchor viewers’ vergence. The ring’s position was fixed across trials, with a zero binocular disparity between the ring and the constant rectangular frame. Hence, throughout an experiment, the perceived 3D positions of the rectangular frame and the ring were unchanged.

The procedure in each test trial, shown in Figure 3A, was similar to that in a previous study.^18^ The current study differed mainly in having the RDSs freely viewed in central vision in all trials, so that gaze was not monitored by an eye tracker. After the observer pressed a button to start a trial, there was a pause of 2 s before the RDSs appeared for τ=200 ms for all but two observers who had τ=400 ms instead so that they could perform the task sufficiently well when the RDS was contrast-matched (whether an observer needed this longer τ was determined by their performance in the practice trials). A binocular mask was shown after the RDSs until the observer’s button press to report for the trial. This mask had the same gray background as that in the RDSs and contained randomly generated black and white lines within the area enclosed by the outer circumference of the ring. The mask images in different trials were generated independently.

Each random-dot was a $0.174^{\circ }\times 0.174^{\circ }$ square in visual angle. The disk’s radius was $3.61^{\circ }$, the ring’s inner and outer radii were $3.7^{\circ }$ and $4.7^{\circ }$. For each surface, the disk or the ring, each dot was randomly and equally likely black or white, positioned randomly on the surface with a spatially uniform probability. This probability was such that the dots would occupy 25% of the image area for each surface if there was zero overlap between different dots. Each dot in one monocular image corresponded to a dot in the other monocular image. This correspondence was contrast-matched for the ring, and was contrast-matched or contrast-reversed for the disk depending on the stimulus condition.

In each trial, the disparity of the disk (and thus of all the disk dots) relative to the ring was equally likely to be positive or negative $0.087^{\circ }$. This made the disk appear in front of or behind the ring if the RDS was contrast-matched. The disk’s binocular disparity was generated by deviating the center of the disk from the center of the ring by $0.087^{\circ }/2$ in opposite directions horizontally in the two monocular images. In a dynamic RDS trial, the disk in all the RDS frames had the same binocular disparity and the same inter-ocular correspondence rule (contrast-matched or contrast-reversed). Within any 200 ms time window, different RDS frames (Δt=10 ms for each frame) differed from each other only in the spatial locations and colors (black and white) of the random dots which were generated independently between the frames. Different trials also involved independently generated random dots. An experimental session contained 200 trials, randomly interleaving trials of different stimulus conditions (2 (dynamic or static) x 2 (contrast-matched or contrast-reversed)), with 25 or 75 trials for each contrast-matched or contrast-reversed RDS condition, regardless of whether the condition was dynamic or static, see Figure 3A.

Figure 3B shows two example RDSs that share the same disk disparity; the upper one is contrast-matched, and the lower one is contrast-reversed. The disparity makes the disk appear in front of the ring in the contrast-matched RDS. Hence, in the contrast-reversed RDS, the reversed depth illusion makes one perceive the disk as being behind the ring. If one free fuses this lower RDS (with gaze directed to this RDS), typically one cannot judge whether the disk is in front of or behind the ring. If one directs the gaze to the upper RDS and free fuses while trying to perceive the lower RDS in the lower peripheral visual field, one is typically better able to perceive the reversed depth. However, in the main experiment, observers viewed the RDS naturally in their central visual field.

Before taking data on the test trials, each observer practiced typically ten static and ten dynamic contrast-matched RDS trials, in order to become familiar with the experimental procedure. In the instructions before the test trials, each observer was told that the test trials included scene images that were noisy so that the depth order could be difficult to judge, and that they should nevertheless report their best guess on the depth order. Each observer was shown several examples of noisy static RDSs for 200 ms, in which various percentages (e.g., 70% and 30%) of the disk dots were contrast-matched, while the other disk dots were noise dots that were independent between the two eyes (i.e., without any statistical correlation in their spatial locations or contrast polarities). These are like the noisy RDSs used in a previous study.^28^ Hence, the naive observers expected challenging trials, but were not aware of the contrast reversal of the disk dots between the eyes in the actual test stimuli. Each observer performed the 200 test trials in a session, with a short break after the 100 th test trial.

Thirteen observers (three male) completed the main experiment. Their ages ranged from 18 to 60 (mean age: 27.6). One observer (the author) was aware of the experiment’s purpose, and two (including the author) had substantial previous experience in judging the depth order of surfaces depicted in contrast-matched and contrast-reversed RDSs. Figure 3C shows the task performance accuracies $F_{a,b}$ for individual observers as well as their averages. When the RDSs were contrast-matched, the average accuracy was near 100% regardless of whether the RDS was static or dynamic.

However, the accuracy for the contrast-reversed RDSs was strongly dependent on whether the RDS was static or dynamic. For the static trials, the observer-averaged accuracy was $F_{\text{reversed},\text{static}}=0.482\pm 0.019$, statistically equivalent (p=0.18) to the chance level of 0.5. However, for the dynamic trials, the accuracy became $F_{\text{reversed},\text{dynamic}}=0.286\pm 0.029$, substantially and significantly (p=0.0001) lower than the chance level. More importantly, averaged across observers, this accuracy was significantly (p=0.0001) lower (therefore better visibility of the reversed depth) than that in the static trials. In each observer, the dynamic accuracy was at least 0.08 lower than the static accuracy. The difference between the dynamic and static accuracies was significant (p≤0.045) in eleven out of our thirteen observers. In other words, most of our observers were significantly better at perceiving the illusion in the dynamic trials compared to the static trials.

The data points for the two observers experienced with contrast-reversed RDSs are marked by “×” (the author) and “⋄” (the naive experienced observer) in Figure 3C. They were better than the other observers at seeing the reversed depth in both the static and the dynamic trials, performing significantly better than chance to see the illusion even in the static trials (p=0.01). (Indeed, a previous study showed that humans can learn to see the reversed depth through practice.^34^) Notably, each of them also saw the illusion significantly better in the dynamic trials than in the static trials (p≤0.0003). Excluding data from these two observers does not qualitatively change the conclusion of the main experiment.

The accuracies in our static RDS condition (both contrast-matched and contrast-reversed) align with previous findings^23^^,^^24^^,^^29^ (including our own previous study,^18^ which used the same equipment setup but with gaze monitoring) that human observers could not see the reversed depth illusion in contrast-reversed RDSs in typical central viewing situations, and when there is no sufficiently effective backward masking to compromise the feedback query. Our dynamic RDS condition, using Δt=10 ms, gave the new finding that the reversed depth illusion becomes visible in central vision when the feedback query is compromised, confirming a key prediction from the CPD theory.

### Central vision resembles peripheral vision in seeing the illusion when the RDSs are more dynamic

Let the feedback queries arrive at an upstream stage of the visual processing pathway at time δt after the initial visual inputs arrive at this stage. In a dynamic trial, if δt<Δt, the queries arrive when the original visual inputs are still available for the queries. However, if δt>Δt, the queries arrive after the original RDS frame has been replaced by the succeeding RDS frames. Although the succeeding and the original frames shared the same (real or illusory) disparity for the disk, the locations and polarities of the dots making up the RDS frames have changed. Hence, for a query to effectively verify whether the inter-ocular correspondence of a particular pair of dots is sensical, it must arrive when the original dots are still present. Such a query requires δt<Δt. Our main experiment suggests that δt>10 ms. To determine this δt more precisely, the secondary experiment added additional Δt=20 ms, 40 ms, 50 ms, and 100 ms, randomly interleaved across trials.

The CPD theory predicts that the reversed depth illusion is more visible when Δt is smaller to compromise the feedback query. Furthermore, it hypothesizes weaker or absent feedback queries at more peripheral visual locations. Indeed the illusion has been observed to be visible at a peripheral visual location but not at a more central visual location in our previous study,^18^ when only large Δt=100 ms was applied. Thus, the CPD theory predicts that as Δt becomes smaller, the illusion should be visible at both the central and peripheral locations, and the visibility difference between these locations should diminish. Hence, the secondary experiment included trials with RDSs viewed at the central and peripheral locations used in the previous study.^18^

There is no clear visual eccentricity boundary to separate the central from the peripheral visual locations, or near-peripheral from (just) peripheral visual locations. We use the terms peripheral and near-peripheral interchangeably, following existing terminologies. Although the CPD theory hypothesizes weaker feedback query at greater eccentricities, it does not specify the minimum eccentricity (if any) required for zero feedback query. Although our previous study^18^ discovered that the reversed depth illusion was seen at a peripheral location, this location’s eccentricity may not have been sufficient to make the feedback query completely absent. To be concrete, let us denote the effectiveness of the feedback query by $Q^{\text{feedback}}$, and let $Q_{\text{central}}^{\text{feedback}}$ or $Q_{\text{peripheral}}^{\text{feedback}}$ denote this quantity at the central or peripheral location in that study. Since that study observed the illusion only at the peripheral location, it implies $Q_{\text{central}}^{\text{feedback}}>Q_{\text{peripheral}}^{\text{feedback}}$ and that $Q_{\text{peripheral}}^{\text{feedback}}$ was too small for an effective feedback veto. However, we cannot rule out a residual $Q_{\text{peripheral}}^{\text{feedback}}>0$. If $Q_{\text{peripheral}}^{\text{feedback}}>0$, reducing Δt should further reduce $Q_{\text{peripheral}}^{\text{feedback}}$ through backward masking, making the illusion even more visible. Meanwhile, if $Q_{\text{peripheral}}^{\text{feedback}}=0$, reducing Δt merely reduces $Q_{\text{central}}^{\text{feedback}}$ and should not affect the visibility of the illusion at the peripheral location. In either case, the CPD theory predicts that reducing Δt will decrease $\left|Q_{\text{central}}^{\text{feedback}}-Q_{\text{peripheral}}^{\text{feedback}}\right|$, either because both $Q_{\text{central}}^{\text{feedback}}$ and $Q_{\text{peripheral}}^{\text{feedback}}$ approach zero by the backward masking, or because $Q_{\text{central}}^{\text{feedback}}$ approaches zero while $Q_{\text{peripheral}}^{\text{feedback}}$ remains at zero. Consequently, the difference in the visibility of this illusion between the central and peripheral locations of the previous study^18^ is predicted to diminish as Δt becomes smaller.

The experimental procedure for the secondary experiment was similar to that of the main experiment, with the following key differences. First, a binocular, zero-disparity, red fixation cross was added to the stereograms. This fixation cross, composed of a vertical bar and a horizontal bar on the gray background, occupied a square size of 0.44°×0.44°. It appeared at the beginning of each trial before the observer pressed a button to initiate the trial. Observers were instructed to fixate on the cross when pressing the button to start a trial and to maintain fixation throughout the RDS presentation duration until the mask appeared. For the central trials, this fixation cross was centered between the inner and outer radius of the ring above the disk. For the peripheral trials, this cross was above the ring at $10.1^{\circ }$ above the disk center. In either case, the fixation cross was in the same depth plane as the ring. These stimulus eccentricities for the central and peripheral trials were the same as those used in the previous study,^18^ which employed a similar procedure.

Each observer was therefore subject to 24 stimulus conditions involving six Δt values (10ms, 20ms, 40ms, 50ms, 100ms, and τ, the last one for static trials), two fixation locations (central and peripheral), and two RDS types (contrast-matched and contrast-reversed). Each observer performed a total of 480 trials, with 10 trials per contrast-matched condition and 30 trials per contrast-reversed condition. Observers took breaks to rest between test blocks, each block lasting about 10–20 minutes.

**Figure 4:**
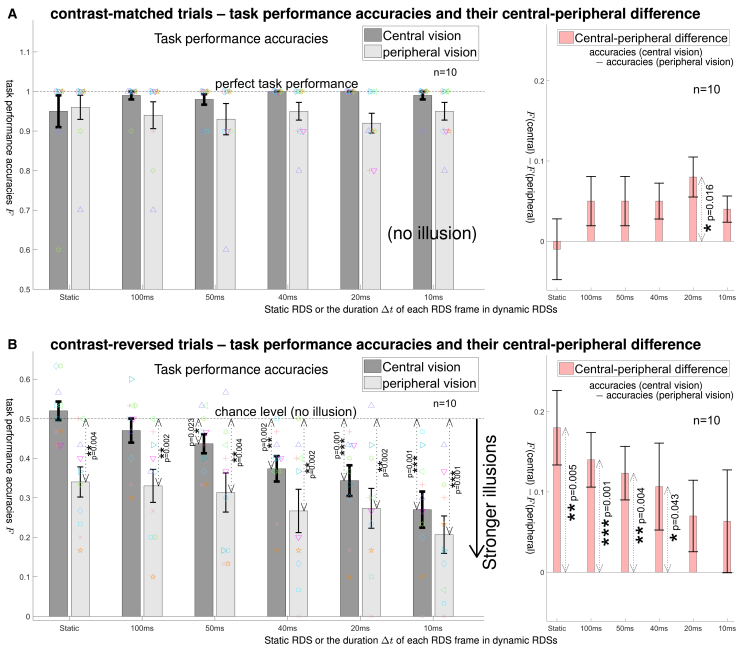
The secondary experiment: decreasing Δt (making RDSs more dynamic) makes the reversed depth illusion more visible and reduces the central-peripheral difference in the visibility of this illusion (A and B) The left panels show the accuracies (F, as defined in the main text or Figure 3) of the depth order reports for each condition: contrast-matched or contrast-reversed, central or peripheral viewing location, and specific Δt or static. The right panels show the difference between the central and peripheral accuracies. (A and B) are for contrast-matched and contrast-reversed RDSs. Bars are the averages across n=10 observers (error bars denote their standard errors), colored symbols represent individual observers’ F values (observers marked by × and ⋄ also participated in the main experiment). In B, the likelihood of reporting the reversed depth illusion, which increases with the downward deviation of F from the chance accuracy level of 0.5, was stronger for shorter Δt. When the RDS was static, the illusion was absent in central vision but substantial in peripheral vision. As Δt→10 ms, the illusion became more visible in both central vision and peripheral vision. The central-peripheral accuracy difference, F (central)−F (peripheral), was substantial and significant for static RDSs in the contrast-reversed (B) but not in the contrast-matched (A) condition. This central-peripheral difference diminished as Δt became smaller in B but such a trend is absent in A. In B, a data bar linked by a dotted-line with arrowheads to value 0.5 (chance level in the left panel) or value 0 (no difference in the right panel) indicates a significant difference from that value by an accompanying p-value from the permutation test for statistical equivalence. One, two, or three asterisks (∗) indicate p<0.05, p≤0.01, or p≤0.001 by permutation tests, respectively.

There were two versions of this secondary experiment. In the first version, each observer performed the central and peripheral trials in separate experimental blocks. Gaze was not monitored, and the RDS viewing duration was τ=200 ms. Three naive observers and the author completed this version of the experiment for both the central and peripheral blocks. Two of them—the author and one naive observer—also participated in the main experiment and were experienced with depth tasks using contrast-matched and contrast-reversed RDSs (their data are marked by the same symbols, “×” and “⋄”, in Figures 3 and 4). One additional observer withdrew from the experiment because, during the practice trials, his performance on the contrast-matched peripheral trials was at the chance level.

Another six naive and inexperienced observers performed the second version of the secondary experiment. In this version, central and peripheral trials were randomly interleaved in the same experimental block. Four of these observers had their gaze monitored by an eye tracker. The tracker’s verification of the proper fixation was required before the RDSs appeared after the 2-s pause (between the observer’s button press to start the trial and the RDS onset). Two of the six observers did not have their gaze tracked (due to tracking difficulties when they wore spectacles). The author visually monitored their fixation behavior using a video camera in most trials and determined it to be acceptable, although occasional fixation breaks occurred almost exclusively during peripheral trials. Because observers often found the task difficult with a short RDS viewing duration τ=200 ms, the second version of the experiment extended this duration to τ=1000 ms. Because the results from the two versions of this experiment were similar, data from all the ten observers (ages ranged from 22 to 59, mean age 40.2, four male) were pooled for analysis.

Before the test trials, each observer had two to four practice trials of each stimulus condition (specified by Δt and the fixation location) of the contrast-matched trials. Like in the main experiment, they were told that some test trials were noisy and difficult, and were shown some examples of noisy, contrast-matched, trials, so that they expected challenging test trials. They were instructed to give their best guess of the disk’s depth order relative to the ring.

Figure 4 shows the accuracies and the difference between the accuracies for the central and peripheral conditions in this experiment. Repeated ANOVA were carried out separately for the contrast-matched and contrast-reversed conditions. Each repeated ANOVA examined the effects of two factors, Δt and viewing eccentricity (central or peripheral). For the contrast-matched RDSs, there was a significant main effect of the viewing eccentricity (F(1,45)=6.13, p=0.0171) but not of Δt (F(5,45)=0.145 and p=0.9805). There was also no significant interaction between Δt and the viewing eccentricity (F(5,45)=0.47, p=0.80). Unsurprisingly, when the RDSs are sensical, central vision is better than peripheral vision in this depth discrimination task. Meanwhile, average task accuracies for such RDSs exceeded 90% in both the central and peripheral conditions, demonstrating that our stimuli had a sufficient spatial resolution even for the peripheral conditions. The Δt value had no significant effect on depth perception of our contrast-matched RDSs. This is consistent with the predictions shown in Figures 1C and 2B that veridical depth perception in contrast-matched RDSs occurs with or without the feedback query.

The contrast-reversed trials gave very different findings. The main effect of the viewing eccentricity was much stronger, with an F-ratio F(1,45)=20.45 and an p-value p<0.0001. The observer-averaged accuracies were below the chance level in each peripheral condition (p≤0.004), indicating the visibility of the illusion. In contrast, the accuracies in the central conditions were statistically equivalent to the chance level for large Δt>50 ms, consistent with the previous finding^18^ that the illusion is only visible in peripheral vision. Meanwhile, for Δt≤50 ms, the central accuracies became significantly below the chance level, the p-values by the permutation test for statistical equivalence to the chance level decreased from p=0.023 for Δt=50 ms to p≤0.002 for Δt≤40 ms. That the illusion became visible in central vision when Δt is small is consistent with the findings from the main experiment.

Furthermore, whereas Δt had no effect on the accuracies in the contrast-matched trials, it had a significant main effect in the contrast-reversed trials, with F(5,45)=5.15 and p=0.0008. Focusing on the contrast-reversed trials, we examine $F_{\text{reversed},\text{static}}-F_{\text{reversed},\Delta t=10\text{ms}}$, the difference between the accuracy ($F_{\text{reversed},\text{static}}$) in the static trials and that ($F_{\text{reversed},\Delta t=10\text{ms}}$) in the most dynamic trials. This difference was highly significant for both the centrally (p=0.001) and peripherally (p=0.012) viewed RDSs. Hence, backward masking increased the visibility of the illusion at both the central and the peripheral locations.

In addition, for each Δt and contrast matching rule, we examine the difference, $F_{a,\Delta t}\left(\text{central}\right)-F_{a,\Delta t}\left(\text{peripheral}\right)$ (a=contrast-matched or a=contrast-reversed), between the accuracy for central vision and that for peripheral vision. For the contrast-reversed RDSs, this central-peripheral difference was substantial and significant for static RDSs (p=0.005), it remained so for dynamic RDS trials using Δt=100 ms (p=0.001) and Δt=50 ms (p=0.004), but this central-peripheral difference became insignificant for small Δt=20 ms and 10 ms, see the right panel of Figure 4B. The ability to see the illusion in central vision started to become significantly better (p=0.023) than chance at Δt=50 ms, and this ability became even more significant (p=0.001) when Δt≤20 ms. There is thus a clear trend that central vision became more and more similar to peripheral vision in its ability to see this illusion as Δt became smaller. This trend of decreasing central-peripheral difference $F_{a,\Delta t}\left(\text{central}\right)-F_{a,\Delta t}\left(\text{peripheral}\right)$ with decreasing Δt was absent in the contrast-matched RDSs (Figure 4A, right). As in the contrast-matched trials, repeated ANOVA revealed no significant interaction between the viewing eccentricity and Δt in the contrast-reversed trials (F(5,45)=0.51, p=0.77).

Hence, using smaller Δt in our dynamic RDSs to increase the impairment of the feedback query, our secondary experiment confirmed the prediction that, when the feedback query is compromised by effective backward masking, central vision becomes statistically equivalent to peripheral vision in the visibility of the reversed depth illusion. Furthermore, it found that, at our eccentricity for viewing the RDS peripherally, there is a significant residual level $Q_{\text{peripheral}}^{\text{feedback}}$ of the feedback query, which is susceptible to further reduction by backward masking, thereby enhancing the illusion.

Our data suggest that at Δt≲20to40 ms, the central-peripheral difference becomes statistically insignificant and the reversed depth illusion becomes reliably visible in central vision. This suggests that 20to40 ms may be the time δt needed by the visual pathway to implement the top-down feedback query. This δt value is consistent with the 30to40 ms latency observed between the feedforward and feedback components of visual cortical responses in monkeys.^35^^,^^36^^,^^37^^,^^38^ The 40 ms duration is also similar to the time lags observed between a target and a mask to produce effective visual backward masking.^26^^,^^27^^,^^39^

Inter-observer variability in task accuracy was greater in the secondary than the main experiment. The following factors likely contributed to this difference. First, the requirement to maintain fixation during the task in the secondary experiment (unlike in the main experiment) increased the demands on observers. Second, the random interleaving of many more different conditions in the secondary experiment (including changes in fixation location across trials for 60% of the observers) further increased task demands. Third, the secondary experiment used fewer trials per condition (10 for contrast-matched and 30 for contrast-reversed) than the main experiment (25 and 75, respectively), which likely increased the noise in accuracy estimates. Nevertheless, despite the difference in fixation requirements, consistent results were found for the conditions common to both experiments: static and Δt=10 ms central vision trials. Similarly, despite the difference in τ, consistent results were found for the four conditions common to the secondary experiment and the previous study^18^: central and peripheral trials with Δt=100 ms. As in the main experiment, removing the data from the two experienced observers (symbols × and ⋄ in Figure 4) in the secondary experiment does not qualitatively change the conclusions. These two observers were the only ones participating in both the main and secondary experiments. Each of these two observers gave statistically equivalent (p≥0.21) performance accuracies in each of the four common conditions (central vision, static or dynamic, Δt=10 ms, contrast-matched or contrast-reversed) for the two experiments, except for the static contrast-reversed condition (p=0.014) for the observer with the data symbol ⋄.

## Discussion

### Summary of the findings and their immediate implications

The CPD theory is falsifiable, since it provides precise, non-trivial, and falsifiable predictions. Two such critical predictions, illustrated in Figures 2C and 2D, are: first, the reversed depth illusion is visible in peripheral, but not central, vision; second, compromising the top-down feedback query makes the illusion visible in central vision, which consequently resembles peripheral vision in the visibility of this illusion. A previous study^18^ confirmed the first prediction. However, this first prediction could arise from explanations alternative to the CPD theory. One alternative is that the higher spatial acuity in central vision somehow makes central vision less susceptible to this illusion. This study confirms the second prediction, thus arguing against the alternative explanation. Therefore, the availability of the top-down feedback query, rather than the higher spatial acuity, is essential for overcoming the illusion. This is consistent with the previous finding that the central-peripheral difference in seeing the reversed depth is insensitive to variations in the sizes of the dots, the disk, the ring, the fixation cross, and the disparity difference between the disk and the ring.^18^

This study compromised the feedback query by backward masking using dynamic RDSs, so that each RDS frame is backward masked by the next RDS frame or another mask Δt later. Our secondary experiment found that, even in peripheral vision which can see the illusion in a static RDS, masking with small Δt=10 ms made the illusion even more visible (Figure 4B). This suggests that the feedback query ($Q_{\text{peripheral}}^{\text{feedback}}$) at this peripheral location was non-zero in the static condition, allowing backward masking to weaken the feedback query further. Meanwhile, this query ($Q_{\text{peripheral}}^{\text{feedback}}$) must be sufficiently weaker than that ($Q_{\text{central}}^{\text{feedback}}$) at the central location, so that the illusion in a static RDS is visible and only visible at the peripheral location. Our peripheral RDS was viewed with the gaze’s position at $10.1^{\circ }$ from the disk’s center, or $6.5^{\circ }$ from the depth edge between the disk and ring. The CPD theory predicts that the feedback query should be even weaker when the RDS is placed at even more eccentric visual locations. It will be valuable to identify the eccentricity at which the feedback query ($Q_{\text{peripheral}}^{\text{feedback}}$) becomes zero. This eccentricity is likely task-dependent, beyond this eccentricity, for example, the visibility of the reversed depth illusion should be unaffected by backward masking.

This study identified that the effect of the backward masking is significant when Δt≲40 to 50 ms, so that the reversed depth illusion becomes visible in central vision. As the backward masking should be effective when Δt<δt, the latency between the feedforward signals and the feedback queries, this Δt≲40 to 50 ms provides a clue about the mechanisms underlying the feedback query. It is consistent with the 30 to 40 ms latency observed between the feedforward and feedback components of visual cortical responses in monkeys.^35^^,^^36^^,^^37^^,^^38^ Considering the response latencies of various visual stages along the visual pathway,^40^^,^^41^ Δt≲40 to 50 ms is consistent with V2 and V4 being among the potential sources of the feedback queries to V1. However, the continued increase in the illusion’s visibility in central vision as Δt→10 ms suggests further complexities. Considering that the feedback query may occur through multiple iterations of the feedforward-feedback loop,^42^ that there can be multiple sources of the feedback queries, and that there can be multiple upstream stages along the visual pathway to receive and support the queries, our data are primarily useful for constraining possible mechanisms involved.

### Relationship between the CPD theory and some previous ideas

Computationally, analysis-by-synthesis is implemented by the feedback from later to earlier stages along the visual pathway to query for additional information for visual decoding.^9^^,^^43^ To infer visual objects from visual input signals, analysis-by-synthesis synthesizes the would-be visual input signals for any perceptual hypothesis, i.e., any candidate outcome of the inference; the degree of the match between the would-be and the actual input signals is then determined to evaluate the suitability of the perceptual hypothesis for perceptual outcome. This is a form of hypothesis testing during visual inference.

Hypothesis testing by top-down feedback for perceptual inference, and related ideas, has been around for many years.^1^^,^^2^^,^^3^^,^^4^^,^^5^^,^^6^^,^^7^^,^^8^^,^^9^ The new contribution by the CPD theory is the hypothesis that this feedback is stronger in the central than the peripheral visual field. Furthermore, the CPD theory uniquely motivates this feedback query by the existence of an information bottleneck that limits the amount of sensory input information that can undergo deeper processing.^10^ If the bottleneck was absent, allowing the transmission of all sensory input information to downstream stages along the visual pathway, there would be no need to query for additional information about the visual inputs from upstream stages. In addition, the CPD theory uses the bottleneck to motivate the distinctive functional roles of peripheral and central vision: peripheral vision is mainly for looking, whereas central vision is mainly for seeing.^10^ Looking selects the tiny fraction of visual input information that can pass the bottleneck (often by deciding on where to shift the gaze to); whereas seeing decodes (i.e., recognizes or infers) visual object properties in the selected information. Central vision is better at seeing by having more resources for the feedback query.

The CPD theory is additionally motivated by the hypothesis that the information bottleneck starts from V1’s output to downstream stages along the visual processing pathway.^10^ This hypothesis makes explicit early selection by visual attention. Based on established knowledge of V1 neural responses to visual inputs,^13^^,^^44^ including the reversed depth responses to contrast-reversed RDSs,^22^ and assuming the bottleneck starting from V1, the CPD theory generates concrete and falsifiable predictions. In particular, the lack of monocular and eye-of-origin information beyond V1^20^ predicts that the reversed depth illusion could arise if perception occurs without the feedback to query for monocular information to invalidate the nonsensical nature of the reversed depth responses from V1’s depth tuned neurons. Furthermore, the feedback query must target V1 (rather than, for example, V2) to retrieve the monocular information for effectively vetoing the reversed depth illusion. This veto is an explicit example of how analysis-by-synthesis works in visual decoding.

The feedback query for additional information from V1 can be related to the idea that V1 (or even the thalamus) serving as a “blackboard” for visual inference or imagery.^45^^,^^46^^,^^47^^,^^48^^,^^49^ These ideas are often inspired by neural anatomy, motivated by the need for a hierarchical, compositional, and/or combinatorial representation of object information, or by a need to facilitate communications between later sensory stages for computations such as feature bindings. The CPD theory in contrast is motivated by the brain’s bottleneck to process sensory information. Since the bottleneck is hypothesized to start from V1’s output to downstream areas along the visual pathway,^10^ V1 has richer information absent in downstream areas.

Our use of Δt≤50 ms in RDSs for backward masking impaired the feedback query, presumably without affecting the frame-invariant feedforward signals about the depth of the disk across our RDS frames within a trial. Our finding that visual perception is differently affected by different Δt=10, 20, 40, 50, and 100 ms suggests that we should re-evaluate the idea to characterize core object recognition, defined as visual recognition in the central $10^{\circ }$ visual field within a limited time of 200 ms, by a model of feedforward neural network of multiple layers, even though such models have showed a remarkable alignment with behavioral, anatomical, and physiological data.^50^^,^^51^ It is indicative that while the ability of such models to predict responses of IT neurons increased with increasing capabilities of these models in object recognition, this ability starts to decrease once the model’s performance in object recognition is better than a turning point.^51^ Two important features of human recognition are missing in such neural network models: one is the information processing bottleneck starting from V1, and the other is the feedback query that favors foveal visual locations. This foveal focus of the feedback query is likely why IT neurons tend to prefer foveal input locations despite having large receptive fields.^52^^,^^53^ Meanwhile, evidence is emerging for feedback to critically help the challenging types of object recognition that are difficult for feedforward neural networks.^54^^,^^55^

### Analysis-by-synthesis via the Feedforward-Feedback-Verify-reWeight (FFVW) algorithm

The steps of the analysis-by-synthesis can be paraphrased as Feedforward-Feedback-Verify-reWeight (FFVW).^17^^,^^42^ First, some V1 signals, r, are *fed forward* to downstream visual areas, suggesting some initial hypotheses about the visual scene. This r can be a low-dimensional function of the high-dimensional V1 responses, the information in r about the external visual inputs has been impoverished by the bottleneck. Second, if the impoverished information in r is insufficient to disambiguate between the alternative perceptual hypotheses about the visual scene, the downstream visual areas synthesize the would-be input signals ${\hat{r}}^{\prime }$ for each hypothesis and *feed* it *back* to earlier visual stages such as V1 to compare with the actual visual input signals $r^{\prime }$. This $r^{\prime }$ is another low-dimensional function of the (e.g.) V1 responses, and should convey additional information that is unavailable from r. The choice to query for $r^{\prime }$, rather than signals from other low-dimensional functions of the V1 responses, should be made such that the additional information from $r^{\prime }$ could best disambiguate between the alternative hypotheses. Third, the comparison between the would-be signals ${\hat{r}}^{\prime }$ and the actual signals $r^{\prime }$ *verifies* the hypothesis. The degree of the match between ${\hat{r}}^{\prime }$ and $r^{\prime }$ is the queried information or an important part of the queried information. Fourth, the initial *weight* associated with the hypothesis is *increased or decreased* if the match is good or poor, respectively.

For example, feature binding^56^ to answer whether a vertical bar should be bound with color red or green can be implemented by the FFVW algorithm to disambiguate between two perceptual hypotheses: hypothesis $H_{1}$ for a red-vertical bar and hypothesis $H_{2}$ for a green-vertical bar.^10^ This feedback query in FFVW can be seen as being operationally similar to looking up information from a blackboard. Next, we explain how FFVW accounts for depth perception in our RDSs.

### CPD predicted illusions by imposing an information bottleneck on feedforward V1 signals

**Figure 5:**
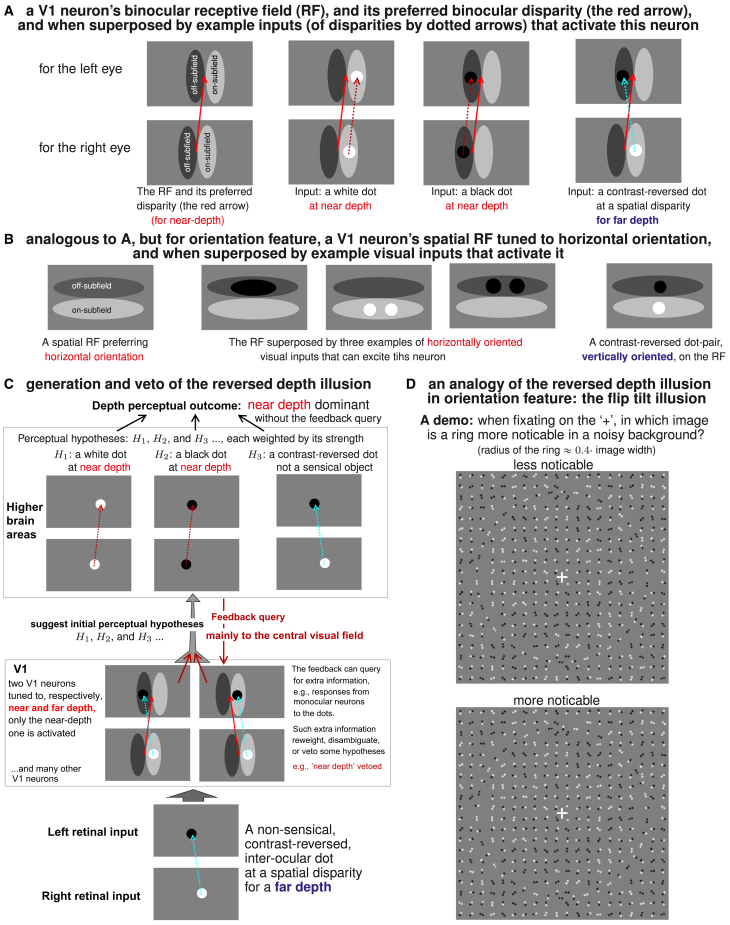
V1 mechanisms and the information bottleneck explain reversed feature illusion in depth and orientation (A) A V1 neuron’s disparity selectivity for encoding depth makes the neuron respond to anti-preferred disparity when the visual input dot is contrast reversed between the two monocular images. (B) An analogous mechanism for encoding orientation. A V1 neuron preferring an horizontal orientation can be excited by a vertically oriented dot-pair of two contrast-reversed dots. (C) Let retinal inputs contain a contrast-reversed dot, with a disparity for a far depth. A V1 neuron tuned to near depth is activated, but not a V1 neuron tuned to far depth. If the bottleneck admits only responses from these two most task-relevant V1 neurons, this impoverished information leads to the reversed depth illusion. Extra information about, e.g., responses of some monocular neurons, queried by the feedback, can veto the illusion. (D) A demonstration of the flip tilt illusion, the analogy of the reversed depth illusion, in peripheral vision but not central vision. In each image, the ring is centered on the “+”, with a radius about 0.4 of the image width. In the upper and lower images, the contrast-reversed dot pairs on the ring are parallel and orthogonal to the ring, respectively.

The reversed depth illusion is an indicative example for testing the CPD theory. Meanwhile the theory applies more generally. Figure 5 illustrates how this illusion arises from V1 mechanisms, drawing an analogy with another illusion predicted by CPD: the flip tilt illusion, which occurs in orientation rather than depth perception. In a schematic of the receptive fields (RFs) of a binocular V1 neuron (the left panel of Figure 5A), the RF for the left-eye image has the same shape as the RF for the right-eye image, but is displaced horizontally from the right-eye RF by a disparity visualized by the red arrow. This arrow is tilted to the right, indicating that this neuron prefers a positive binocular disparity for a near-depth. The second panel of Figure 5A shows the neural RFs superposed by a contrast-matched white binocular dot, whose disparity is visualized by a dashed red arrow. The white dot falls in the on-subfields of the RFs in both eyes, hence the dot’s disparity matches the preferred disparity and so excites this neuron. The third panel of Figure 5A is analogous to the second panel, illustrating that a contrast-matched black dot at near-depth can also excite this neuron via the off-subfields of the RFs. However, as shown in the right panel of Figure 5A, this neuron can also be excited by a negative disparity (for a far-depth, the left-tilted cyan arrow) opposite to the preferred disparity when the dot is contrast-reversed, because the black dot falls in an off-subfield for one eye while the white dot falls in an on-subfield for the other eye to excite this neuron. This contrast-reversed dot does not activate another V1 neuron tuned to far depth (see Figure 5C). This is a simplified model (see a fuller model in a study by Read J.C.A et al.^57^) to explain the reversed depth responses in real V1.^22^

Figure 5B shows an analogous schematic for a V1 neuron tuned to horizontal orientation. This neuron is excited by horizontally oriented inputs, such as a pair of contrast-matched dots (two white dots or two black dots) horizontally displaced from each other. But this neuron is also excited by a vertically oriented input when the dot-pair is contrast-reversed. This predicts the flip tilt illusion—the perceived orientation of a contrast-reversed dot pair is orthogonal to the actual orientation of this dot pair in the peripheral but not the central visual field.^19^

Figure 5C explains how V1’s reversed depth responses lead to the illusion by a bottleneck starting from V1’s output to later stages. A contrast-reversed dot across the two retinas, with a far-depth disparity, excites a V1 neuron tuned to near-depth but not another V1 neuron tuned to far-depth. Imagine a simple case in which these two most task-relevant V1 neurons’ responses r are the only ones admitted into the bottleneck to downstream visual areas. Based on these responses and the knowledge about these neurons’ RF structures, downstream visual areas generate multiple hypotheses, e.g., $H_{1}$, $H_{2}$, $H_{3}$, about the visual scene that caused r. Hypothesis $H_{1}$ is for a white dot at near-depth, hypothesis $H_{2}$ is for a black dot at near-depth, hypothesis $H_{3}$ is for a nonsensical, contrast-reversed, dot. Therefore, the perceptual outcome is ambiguous between the three possible perceptual outcomes, because the bottleneck has impoverished the feedforward information so that the perceptual process cannot narrow down to a single hypothesis by the admitted signals r only. If (as hypothesized for peripheral vision) there is no option to query for more information from V1, the perceptual process should use its internal model of the world to assign a weight $w_{i}$, or probability $p_{i}$, to each $H_{i}$, leading to a probability $p_{\text{near}-\text{depth}}=p_{1}+p_{2}$ for near-depth and a probability of $p_{\text{nonsensical}}=p_{3}$ for a nonsensical scene. However, the probability $p_{\text{far}-\text{depth}}=0$ for far-depth is zero since r suggests that there is no far-depth object in the scene. Therefore, $p_{\text{near}-\text{depth}}>p_{\text{far}-\text{depth}}$ as long as $p_{1}+p_{2}>0$. Between near-depth and far-depth options, a better guess should be near-depth to maximize the probability of a correct guess, this gives the reversed depth illusion. Downstream visual areas are assumed to have a knowledge about the visual world, so that they know the would-be V1 responses ${\hat{r}}^{\prime }$ from, e.g., monocular neurons for the dot’s monocular locations for each hypothesis $H_{i}$. Through the FFVW process that is available to central vision, the feedback query should find that ${\hat{r}}^{\prime }$ does not match the actual V1 response $r^{\prime }$ for hypotheses $H_{1}$ and $H_{2}$, and thus reweight or veto the reversed depth illusion by making $p_{1}=p_{2}=0$.

If the information bottleneck were absent, downstream visual areas would have access to all information encoded by V1, including monocular information. This would be true even if the monocular information were encoded implicitly in the population activity of downstream neurons rather than explicitly by dedicated monocular neurons. In this scenario, downstream areas could decode the necessary monocular information directly from their population activity. Consequently, the reversed depth illusion could be vetoed without requiring a feedback query to V1, then the reversed depth illusion would not occur.

The flip tilt illusion arises in a manner analogous to that explained in Figure 5C for the reversed depth illusion.^19^ Figure 5D demonstrates the flip tilt illusion by two images containing many contrast-matched and contrast-reversed pairs of dots. All the dot-pairs are randomly positioned and oriented except those on a ring centered on the central white cross in each image. On this ring, alternating dot-pairs are either contrast-matched and tangential to the ring, or contrast-reversed. In the upper image, the contrast-reversed dot-pairs on the ring are also tangential to the ring. In the lower image, the contrast-reversed dot-pairs on the ring are orthogonal to the ring. When the image is viewed casually or with gaze fixated on the central cross, the ring is primarily in the peripheral visual field. In this case, the ring is much more noticeable against the noisy background in the lower image than in the upper image. This is because the flip tilt illusion makes the contrast-reversed pairs appear orthogonal to their actual orientations in the peripheral visual field. Consequently, in the lower ring, all its dot-pairs appear tangentially aligned with the ring to make the ring more noticeable. In contrast, in the upper ring, only its contrast-matched dot-pairs appear aligned with the ring, making the ring less noticeable. When gaze is directed to the ring and examines the ring in central vision, the flip tilt illusion is vetoed and the upper ring (or at least the ring segments near the center of gaze) is more noticeable.

Another illusion analogous to the reversed depth illusion is the reversed phi motion illusion (of the motion direction), which is also stronger in peripheral than central vision,^58^^,^^59^ and can be understood analogously.^19^ In general, these reversed feature illusions suggest that the reversed depth illusion is not functionally special, despite the rather transparent loss of eye-of-origin information from V1 to V2 along the visual pathway. Because orientation and motion direction features are encoded beyond V1, the existence of the corresponding reversed feature illusions suggests that the bottleneck operation progressively loses critical information along the visual pathway. In other words, although the bottleneck starts from V1’s output to downstream areas, information loss continues in downstream areas. Cumulatively, more and more information is lost as one travels further downstream along the visual pathway. Hence, the downstream visual areas beyond V1 are likely to have a hierarchical organization for the feedforward and feedback operations in visual inference, such that the feedback query could be sent from a relatively downstream node in the hierarchy to a relatively upstream node that may not be V1.

### Beyond illusions

The reversed depth illusion (and other reversed feature illusions) and how its visibility changes with visual input locations or backward masking are only some of the falsifiable predictions of the CPD theory. The theory can be tested by its other predictions. One prediction is that the effect of backward masking on visual discrimination (rather than illusion) should be weaker at more peripheral visual locations.^10^ This prediction has been confirmed in an example of metacontrast visual masking,^60^ and can be tested further in other examples of visual discrimination tasks, especially when the discrimination performance is sufficiently sensitive to visual backward masking. Another prediction, via CPD’s hypothesis that central and peripheral vision are mainly for seeing (recognition) and looking (shifting attention), is that visual saliency has a stronger effect to guide attention exogenously at more peripheral visual locations. This prediction has also been confirmed in a visual search task,^61^^,^^62^^,^^63^ and can also be tested further by other examples of visual search tasks. Neurally, the CPD theory predicts that the reversed depth signals (or signals for other related illusions) in neural responses are stronger in neurons whose receptive fields cover more peripheral parts of the visual field in various visual cortical areas. These visual areas are more likely to be in visual areas downstream of V1 along the visual pathway, and may also include V1 if the feedback query also affects V1 responses. It also predicts that these reversed feature signals in neural responses are likely stronger sooner after the visual input onset, before the effects of the feedback query have fully developed. Another neural prediction of the CPD theory is that, in the early stages, such as V1 and V2, along the visual pathway, the percentage of the neurons that are simple cells (rather than complex cells) should be higher at cortical locations representing the more central rather than the more peripheral visual locations. This is because responses from simple cells are more sensitive to spatial locations of the visual inputs, they should therefore have more information about spatial details of visual inputs to support the feedback query, which is assumed to be stronger in the central visual field by the CPD theory. This prediction can be easily tested. Anatomically, one should also test another CPD prediction that, in the ventral visual pathway for visual recognition, there should be denser top-down feedback fibers targeting the central than the peripheral visual field representations in upstream cortical areas such as V1.

Feedback to representations of the central visual field can not only veto, confirm, or disambiguate between perceptual hypotheses, it can also exert top-down biases to perception. For example, a previous study^17^ showed that, during ambiguous perception, there is a bias to perceive orientation, color, or motion direction in the binocular summation channel rather than the dichoptic contrast channel (these two channels are represented in V1 responses^64^^,^^65^). This bias is in our brain’s internal model used for the analysis-by-synthesis computation, and, because feedback query is used to implement analysis-by-synthesis, this bias is stronger in the central visual field.^17^ The feedback to aid visual recognition can also act on sensory inputs in a nonlinear, context-dependent, and constructive manner. For example, a hybrid RDS containing both contrast-matched and contrast-reversed dots can depict 3D object surfaces. If these dots share the same binocular disparity (of the 3D surface), then, as long as the RDS is viewed in central vision for a sufficiently long duration for the feedback query to function adequately, the perceived depth surface is as if the contrast-reversed dots were vetoed and treated as noise. However, if the viewing is too brief (e.g., 20 ms) for the feedback to take effect, depth perception is impaired as if the contrast-reversed dots undermine perception more strongly than mere noise.^28^ On the other hand, if the contrast-reversed dots and the contrast-matched dots have opposite disparity values, so that their evoked signals in V1 agree with each other on the depth sign, then the contrast-reversed dots augment depth perception of the 3D surface regardless of the viewing duration.^28^ This perceptual augmentation by contrast-reversed dots may be related to top-down visual imagination to see vivid subjective contours for a (e.g.) Kanizsa triangle using fragmentary and imperfect visual inputs.^66^^,^^67^ Without visual inputs, top-down imagination is likely active during wakeful rest, predicting via the CPD theory a correlation between neural activities in higher brain areas with those in the foveal region of V1. This is consistent with the finding that angular gyrus, a brain region associated with such functions as language and arithmetics, has its neural activities more strongly correlated with foveal V1 activities during rest without visual inputs than during movie watching^68^ when bottom-up feedforward visual inputs contribute additionally to drive neural activities. We are only at the beginning of exploring and testing the visual perceptual framework of seeing through the bottleneck from V1 and the CPD theory.^10^ This will also enable us to examine peripheral vision in a new light in terms of its functional role for looking rather than seeing.^69^

The top-down feedback query to aid seeing can be placed in the framework that vision has the following three computational stages^13^: encoding → selection ⇌ decoding. This framework centers on the selection stage, which selects a tiny fraction of encoded visual information into a bottleneck for deeper processing. In this framework, the selected inputs are sent forward to be decoded, the decoding process can influence selection through feedback. Since selection often involves a gaze shift to place the selected location or object at fovea, the feedback query about an object initially at a peripheral location before the gaze shift should be directed to the expected foveal location of this object after the gaze shift; this means some feedback can be from a peripheral visual location (represented in a downstream cortical stage) to a foveal location (represented in an upstream stage along the visual pathway).^70^^,^^71^ Regardless of whether the selection is by a gaze shift, across a gaze shift, or during a fixation (accompanied by fixational eye movements^72^), the feedback query involves selecting specific information (contained in $r^{\prime }$) into the bottleneck during the feedback query and during selection by or across gaze shifts. This interplay between selection and decoding is particularly relevant for visual decision-making tasks involving active sampling of the environment, such as foraging or planning.^16^

### Limitations of the study

This study reports behavioral tests of the CPD theory, which focuses on the computational and algorithmic aspects. The theory does not yet specify the neural implementation details of the computation. For example, it does not specify which brain areas are sending the feedback query, other than, qualitatively, that they are downstream of V1 along the visual pathway. It does not yet specify how the FFVW algorithm is implemented in neural circuits, for example, whether and how should the feedback query affect V1 neural activities, or which layers of neurons in a cortical area should receive the top-down feedback. The concrete behavioral phenomena reported here, and their confirmation of the theoretical predictions, should motivate investigations at the neural level to fill in the missing information for the implementation. In particular, the behavioral tasks in this study could be easily adapted for experiments in monkeys by simultaneous behavioral and neural recordings. Established knowledge about how V1 neurons should respond to such visual inputs can help neurophysiological investigations to examine neural responses at multiple stages along the visual pathway. (For example, it is likely easier to find V1 neurons responding to the contrast-matched and contrast-reversed RDSs for depth surfaces, than to find V1 neurons responding to some segments of subjective contours in a Kanizsa triangle or a mooney image, in order to investigate the top-down feedback mechanisms in visual imagery.) Indeed, it has been observed that, while V1 neurons are tuned to binocular disparity in both contrast-matched and contrast-reversed RDSs,^22^ neurons in V4 downstream of V1 are on average less tuned to disparity when the RDS is contrast-reversed.^73^ This is consistent with a veto of the nonsensical V1 signals by downstream processing stages along the visual pathway, and hence one might speculate that V4 is among the areas downstream of V1 to send the feedback query. These studies and future ones to test the neural predictions (mentioned previously) of the CPD theory can hopefully suggest which downstream brain areas are more or less involved in the perceptual decision making, and if and how they should be organized in a hierarchical structure (e.g., to differentiate between V2, V4, and IT downstream of V1). Then, information on the neural mechanisms discovered by the future studies can be combined with the current behavioral findings to build a concrete model of the FFVW process for visual inference, thereby making additional falsifiable predictions to further test the CPD theory. Meanwhile, some supports for the CPD theory at the neural level are emerging. For example, Sims et al.^74^ reported that functional connectivity (through data from functional magnetic resonance imaging) between V1 and frontal cortical areas is stronger in V1 regions representing the central than the peripheral visual field. Also, simultaneous large-scale multielectrode electrophysiological recordings from monkey visual cortex V1 and V4 reveal, through Granger-causality analysis, a stronger top-down feedback from V4 to V1 into foveal rather than more peripheral regions.^75^ To further test and develop the CPD theory, we need investigations using diverse approaches—computational, behavioral, anatomical, and neurophysiological—to get a full understanding.

## Resource availability

### Lead contact

Further information can be available by contacting the author Li Zhaoping at li.zhaoping@tuebingen.mpg.de.

### Materials availability

This study did not generate new unique reagents or other materials.

### Data and code availability

• Data: The data from this study have been deposited in Open Science Framework. The DOI is listed in the key resources table.
• Code: The paper does not report any original code.
• Additional information: Any additional information required to reanalyze the data reported in this paper is available from the lead contact upon request.

## Acknowledgments

This work is supported in part by funding from the 10.13039/501100004189Max Planck Society and the 10.13039/501100002345University of Tübingen. I like to thank Peter Dayan and three anonymous reviewers for comments on this paper.

## Author contributions

L.Z. is the only author of this paper.

## Declaration of interests

The author declares no competing interests.

## STAR★Methods

### Key resources table

**Table:** 

REAGENT or RESOURCE	SOURCE	IDENTIFIER
**Deposited data**		

Raw data	This study	https://doi.org/10.17605/OSF.IO/MXU9W

**Software and algorithms**		

MATLAB	https://www.mathworks.com/	Any recent version

### Experimental model and study participant details

The experiments in this study are part of our research program titled “Investigation of sensory perception (visual, auditory, olfactory, tactile) by human behaviorial experiments”, and this program, under ethical application number 2019_01, has been approved by the Ethics Council of the Max Planck Society.

Thirteen observers (three male, eleven white Europeans, two Asians) completed the main experiment. Their ages ranged from 18 to 60 (mean age: 27.6). Ten observers (ages ranged from 22 to 59, mean age 40.2, four male, seven white Europeans, three Asians) participated in the secondary experiment. All observers were healthy, had normal or corrected-to-normal vision, and gave informed consent before the study. The experiments were performed in accordance with the Declaration of Helsinki, except for preregistration. This study does not analyze whether our findings are specific to the gender and ethnicity of our observers. This is mainly because investigating the specificity to gender and ethnicity is not the purpose of the study, and the numbers of the observers for different genders and ethnicities were not statistically appropriate for such an analysis.

### Method details

Once the designs (in the results section) of the experiments in this study are determined, most of the methods to implement the designs and to analyze the data are standard (university level) procedures in experimental psychology. These procedures include displaying visual stimuli, tracking observers’ gaze, recording observers’ button presses, obtaining the averages and their standard errors from measurements across trials or across observers, and carrying out analyses of variances (ANOVAs).

A somewhat non-standard procedure in the data analysis is to carry out the permutation tests. To determine whether an accuracy $F_{a,b}$, averaged across observers, is statistically equivalent to the chance accuracy of 0.5, a permutation test is performed as follows. Let $F{\left(s\right)}_{a,b}$ be the accuracy by subject s=1,2,...,N of N observers, and let $x\left(s\right)=F{\left(s\right)}_{a,b}-0.5$. The average $\bar{x}\equiv \frac{1}{N}\sum_{s=1}^{N}x\left(s\right)$ is compared with ${\bar{x}}^{\prime }\equiv \frac{1}{N}\sum_{s=1}^{N}{\left(-1\right)}^{b\left(s\right)}x\left(s\right)$, for the binary value b(s)=0 or b(s)=1. Among all the possible ${\bar{x}}^{\prime }$ values from all the $2^{N}$ possible combinations of b(1), b(2)., and b(N), the p-value of the permutation test is the fraction of the ${\bar{x}}^{\prime }$ s that are larger than $\bar{x}$ or the fraction of ${\bar{x}}^{\prime }$ s that are smaller than $\bar{x}$, whichever fraction is smaller. Analogously, to test whether the observer-averaged $F_{a,b}$ and $F_{a^{\prime },b^{\prime }}$ for two different conditions are statistically equivalent, we replace x(s) by $x\left(s\right)=F{\left(s\right)}_{a,b}-F{\left(s\right)}_{a^{\prime },b^{\prime }}$ when using the same testing procedure above. For each subject s, to test whether $F{\left(s\right)}_{a,b}$ and $F{\left(s\right)}_{a^{\prime },b^{\prime }}$ are statistically equivalent (or $F{\left(s\right)}_{a,b}>F{\left(s\right)}_{a^{\prime },b^{\prime }}$ as the alternative hypothesis), we compare $x\left(s\right)\equiv F{\left(s\right)}_{a,b}-F{\left(s\right)}_{a^{\prime },b^{\prime }}$ with $x^{\prime }\left(s\right)\equiv F^{\prime }{\left(s\right)}_{a,b}-F^{\prime }{\left(s\right)}_{a^{\prime },b^{\prime }}$, in which $F^{\prime }{\left(s\right)}_{a,b}$ and $F^{\prime }{\left(s\right)}_{a^{\prime },b^{\prime }}$ are the accuracies obtained after each trial of these two conditions is randomly assigned to condition (a,b) or $\left(a^{\prime },b^{\prime }\right)$ while keeping the total number of trials for each condition intact. The p-value of this permutation test is the fraction of the permutations of trial labels (across all possible or randomly sampled permutations) for which $x^{\prime }\left(s\right)>x\left(s\right)$ is achieved.

### Quantification and statistical analysis

All the reported quantities in this paper have been defined in the main text at their appropriate sections or paragraphs for the ease of reading. For completeness, I summarize them here.

• In our depth reporting task, the accuracy of each observer in each condition (as plotted in Figures 3 and 4) is defined as the ratio between the number of trials of this condition in which the depth report agreed with the disparity of the disk and the number of all the trials of this condition performed by this observer.
• In Figures 3 and 4, each data bar represents the accuracy averaged across n observers, with n=13 and 10 for the main and secondary experiments, respectively. The error bars in these figures represent the standard errors of the average accuracies. These accuracies are also reported in the text in the results section.
• Repeated ANOVA (analysis of variance) is employed to analyze data in the secondary experiment, to examine the effects of two factors: viewing location (central or peripheral) and the duration Δt for each RDS frame. The results of the analysis are shown by F ratios and p-values in the text of the results section.
• Permutation test is used to examine whether one observer-averaged quantity (e.g., accuracies) in one condition is larger than, smaller than, or statistically equivalent to, that in another condition. The same test can also compare the average accuracy with another constant such as the chance level of 0.5, or to examine whether the difference between two accuracies is different from zero. Permutation test is also used to compare two accuracies of the same observer in two different conditions. The detailed procedures for the permutation test are described in the Method details above. The p-values of these permutation tests are reported in the text and the figure captions of the results section.
• All the analysis above could be done by hand, in principle. In practice, for time efficiency, it is recommended to use standard or self-written computer programs using Matlab, Python, or any other suitable language for the analysis.

### Additional resources

No additional resources beyond the standard ones available from, e.g., equipment suppliers and basic research infrastructure, are needed to reproduce this study.

## References

[1] Al-Haytham (1989). The optics of Ibn Al-Haytham books I-III: on direct vision. Translated by A.I. Sabra.

[2] von Helmholtz H. (1925).

[3] MacKay D.M. (1956). Towards an information-flow model of human behavior. Br. J. Psychol..

[4] Carpenter G.A., Grossberg S. (1987). ART 2: Self-organization of stable category recognition codes for analog input patterns. Appl. Opt..

[5] Li Z. (1990). A model of olfactory adaptation and sensitivity enhancement in the olfactory bulb. Biol. Cybern..

[6] Kawato M., Hayakawa H., Inui T. (1993). A forward-inverse optics model of reciprocal connections between visual cortical areas. Netw. Comput. Neural Syst..

[7] Dayan P., Hinton G.E., Neal R.M., Zemel R.S. (1995). The Helmholtz machine. Neural Comput..

[8] Rao R.P., Ballard D.H. (1999). Predictive coding in the visual cortex: A functional interpretation of some extra-classical receptive field effects. Nat. Neurosci..

[9] Yuille A., Kersten D. (2006). Vision as Bayesian inference: Analysis by synthesis?. Trends Cogn. Sci..

[10] Zhaoping L. (2019). A new framework for understanding vision from the perspective of the primary visual cortex. Curr. Opin. Neurobiol..

[11] Sziklai G. (1956). Some studies in the speed of visual perception. IEEE Trans. Inf. Theory.

[12] Kelly D. (1962). Information capacity of a single retinal channel. IEEE Trans. Inf. Theory.

[13] Zhaoping L. (2014).

[14] Simons D.J., Chabris C.F. (1999). Gorillas in our midst: Sustained inattentional blindness for dynamic events. Perception.

[15] Li Z. (2002). A saliency map in primary visual cortex. Trends Cogn. Sci..

[16] Zhaoping L. (2023). Peripheral and central sensation: Multisensory orienting and recognition across species. Trends Cogn. Sci..

[17] Zhaoping L. (2017). Feedback from higher to lower visual areas for visual recognition may be weaker in the periphery: Glimpses from the perception of brief dichoptic stimuli. Vision Res..

[18] Zhaoping L., Ackermann J. (2018). Reversed depth in anticorrelated random-dot stereograms and the central-peripheral difference in visual inference. Perception.

[19] Zhaoping L. (2020). The flip tilt illusion: Visible in peripheral vision as predicted by the central-peripheral dichotomy. Iperception..

[20] Hubel D.H., Wiesel T.N. (1977). Ferrier lecture: Functional architecture of macaque monkey visual cortex. Proc. R. Soc. Lond. B Biol. Sci..

[21] Ono H., Barbeito R. (1985). Utrocular discrimination is not sufficient for utrocular identification. Vision Res..

[22] Cumming B.G., Parker A.J. (1997). Responses of primary visual cortical neurons to binocular disparity without depth perception. Nature.

[23] Julesz B. (1971).

[24] Cumming B.G., Shapiro S.E., Parker A.J. (1998). Disparity detection in anticorrelated stereograms. Perception.

[25] Cogan A.I., Kontsevich L.L., Lomakin A.J., Halpern D.L., Blake R. (1995). Binocular disparity processing with opposite-contrast stimuli. Perception.

[26] Enns J.T., Di Lollo V. (1997). Object substitution: A new form of masking in unattended visual locations. Psychol. Sci..

[27] Enns J.T., Di Lollo V. (2000). What’s new in visual masking?. Trends Cogn. Sci..

[28] Zhaoping L. (2021). Contrast-reversed binocular dot-pairs in random-dot stereograms for depth perception in central visual field: Probing the dynamics of feedforward-feedback processes in visual inference. Vision Res..

[29] Doi T., Tanabe S., Fujita I. (2011). Matching and correlation computations in stereoscopic depth perception. J. Vis..

[30] Hibbard P.B., Scott-Brown K.C., Haigh E.C., Adrain M. (2014). Depth perception not found in human observers for static or dynamic anti-correlated random dot stereograms. PLoS One.

[31] Crick F., Koch C. (1998). Consciousness and neuroscience. Cereb. Cortex.

[32] Doi T., Takano M., Fujita I. (2013). Temporal channels and disparity representations in stereoscopic depth perception. J. Vis..

[33] Zhaoping L. (2012). Gaze capture by eye-of-origin singletons: Interdependence with awareness. J. Vis..

[34] Reeves A., Lynch D. (2017). Transparency in stereopsis: Parallel encoding of overlapping depth planes. J. Opt. Soc. Am. A Opt. Image Sci. Vis..

[35] Chen M., Yan Y., Gong X., Gilbert C.D., Liang H., Li W. (2014). Incremental integration of global contours through interplay between visual cortical areas. Neuron.

[36] Chen R., Wang F., Liang H., Li W. (2017). Synergistic processing of visual contours across cortical layers in V1 and V2. Neuron.

[37] Yan Y., Zhaoping L., Li W. (2018). Bottom-up saliency and top-down learning in the primary visual cortex of monkeys. Proc. Natl. Acad. Sci. USA.

[38] Klink P.C., Dagnino B., Gariel-Mathis M.-A., Roelfsema P.R. (2017). Distinct feedforward and feedback effects of microstimulation in visual cortex reveal neural mechanisms of texture segregation. Neuron.

[39] Di Lollo V., Enns J.T., Rensink R.A. (2000). Competition for consciousness among visual events: The psychophysics of reentrant visual processes. J. Exp. Psychol. Gen..

[40] Schmolesky M.T., Wang Y., Hanes D.P., Thompson K.G., Leutgeb S., Schall J.D., Leventhal A.G. (1998). Signal timing across the macaque visual system. J. Neurophysiol..

[41] Bullier J., Nowak L.G. (1995). Parallel versus serial processing: New vistas on the distributed organization of the visual system. Curr. Opin. Neurobiol..

[42] Zhaoping L. (2025). Vision: Looking and seeing through our brain’s information bottleneck. PsyArXiv.

[43] Kersten D., Mamassian P., Yuille A. (2004). Object perception as Bayesian inference. Annu. Rev. Psychol..

[44] Graham N.V.S. (1989).

[45] Deacon T.W., Perecman E. (1989). Integrating theory and practice in clinical neuropsychology.

[46] Mumford D. (1992). On the computational architecture of the neocortex: II the role of cortico-cortical loops. Biol. Cybern..

[47] Kosslyn S.M., Ganis G., Thompson W.L. (2001). Neural foundations of imagery. Nat. Rev. Neurosci..

[48] Van der Velde F., De Kamps M. (2006). Neural blackboard architectures of combinatorial structures in cognition. Behav. Brain Sci..

[49] Roelfsema P.R., de Lange F.P. (2016). Early visual cortex as a multiscale cognitive blackboard. Annu. Rev. Vis. Sci..

[50] DiCarlo J.J., Zoccolan D., Rust N.C. (2012). How does the brain solve visual object recognition?. Neuron.

[51] Kar K., DiCarlo J.J. (2024). The quest for an integrated set of neural mechanisms underlying object recognition in primates. Annu. Rev. Vis. Sci..

[52] Obara K., O’Hashi K., Tanifuji M. (2017). Mechanisms for shaping receptive field in monkey area TE. J. Neurophysiol..

[53] Op De Beeck H., Vogels R. (2000). Spatial sensitivity of macaque inferior temporal neurons. J. Comp. Neurol..

[54] Tang H., Schrimpf M., Lotter W., Moerman C., Paredes A., Ortega Caro J., Hardesty W., Cox D., Kreiman G. (2018). Recurrent computations for visual pattern completion. Proc. Natl. Acad. Sci. USA.

[55] Kar K., Kubilius J., Schmidt K., Issa E.B., DiCarlo J.J. (2019). Evidence that recurrent circuits are critical to the ventral stream’s execution of core object recognition behavior. Nat. Neurosci..

[56] Treisman A.M., Gelade G. (1980). A feature-integration theory of attention. Cogn. Psychol..

[57] Read J.C.A., Parker A.J., Cumming B.G. (2002). A simple model accounts for the response of disparity-tuned V1 neurons to anticorrelated images. Vis. Neurosci..

[58] Anstis S.M. (1970). Phi movement as a subtraction process. Vision Res..

[59] Anstis S.M., Rogers B.J. (1975). Illusory reversal of visual depth and movement during changes of contrast. Vision Res..

[60] Zhaoping L., Liu Y. (2022). The central-peripheral dichotomy and metacontrast masking. Perception.

[61] Zhaoping L. (2022). Parallel advantage: Further evidence for bottom-up saliency computation by human primary visual cortex. Perception.

[62] Liang J., Maher S., Zhaoping L. (2023). Eye movement evidence for the V1 saliency hypothesis and the central-peripheral dichotomy theory in an anomalous visual search task. Vision Res..

[63] Liang J., Zhaoping L. (2025). Trans-saccadic integration for object recognition peters out with pre-saccadic object eccentricity as target-directed saccades become more saliency-driven. Vision Res..

[64] Li Z., Atick J.J. (1994). Efficient stereo coding in the multiscale representation. Netw. Comput. Neural Syst..

[65] May K., Zhaoping L. (2022). Li and atick’s theory of efficient binocular coding: A tutorial and mini-review. Vision Res..

[66] Kanizsa G. (1976). Subjective contours. Sci. Am..

[67] Bertamini M., Kubovy M. (2006).

[68] Knapen T. (2021). Topographic connectivity reveals task-dependent retinotopic processing throughout the human brain. Proc. Natl. Acad. Sci. USA.

[69] Zhaoping L. (2024). Peripheral vision is mainly for looking rather than seeing. Neurosci. Res..

[70] Williams M.A., Baker C.I., Op de Beeck H.P., Shim W.M., Dang S., Triantafyllou C., Kanwisher N. (2008). Feedback of visual object information to foveal retinotopic cortex. Nat. Neurosci..

[71] Fan X., Wang L., Shao H., Kersten D., He S. (2016). Temporally flexible feedback signal to foveal cortex for peripheral object recognition. Proc. Natl. Acad. Sci. USA.

[72] Rucci M., Poletti M. (2015). Control and functions of fixational eye movements. Annu. Rev. Vis. Sci..

[73] Tanabe S., Umeda K., Fujita I. (2004). Rejection of false matches for binocular correspondence in macaque visual cortical area V4. J. Neurosci..

[74] Sims S.A., Demirayak P., Cedotal S., Visscher K.M. (2021). Frontal cortical regions associated with attention connect more strongly to central than peripheral V1. Neuroimage.

[75] Morales-Gregorio A., Kurth A.C., Ito J., Kleinjohann A., Barthélemy F.V., Brochier T., Grün S., van Albada S.J. (2024). Neural manifolds in V1 change with top-down signals from V4 targeting the foveal region. Cell Rep..

